# Hepatitis B coinfection in HIV-infected patients

**DOI:** 10.1186/1471-2334-13-S1-P13

**Published:** 2013-12-16

**Authors:** Gabriela Jugănariu, Cristina Nicolau, Liviu Jany Prisăcariu, Carmen Manciuc, Andra Teodor, Cătălina Luca, Carmen Dorobăț

**Affiliations:** 1 “Gr.T.Popa” University of Medicine and Pharmacy, Iaşi, Romania; 2 Infectious Diseases Hospital “Sf Parascheva”, Iaşi, Romania

## Background

The similar routes of transmission for HIV and HBV infections place patients with either infection at greater risk for HIV/HBV co-infection. The aim of this study was to analyze the epidemiological, virological, immunological and evolutive characteristics of HIV-HBV coinfected patients in the Regional HIV/AIDS Center Iaşi.

## Methods

We performed a retrospective study including 252 patients diagnosed with HIV associated with hepatitis B virus coinfection evaluated in the Infectious Diseases Hospital Iaşi between 2000-2013.

## Results

HIV-HBV coinfection prevalence was 19.9%. We noticed a slightly higher frequency of coinfection in males (53.2%), most patients belonging to the age group 20-29 (86.5%), the median age of the group being 25.56 years. The predominant route of transmission was parenteral (57.5%), heterosexual being found in a significant proportion (40.1%). The mean CD4 cell count was 246.20 cells/cmm, over 41% of the cases had levels between 200-499 CD4 cells/cmm. Individual values of plasma HIV viral load varied from undetectable levels up to a maximum of 4,600,000 copies/mL, with a median value of 142,906 copies/mL. ALT levels in HIV and HBV coinfection varied between 10-323 U/L, the average being 49.90 U/L, more than 65% of subjects with pathological levels; 218% of the total cholesterol values were pathological, the mean for the group was 182.58 mg/dL. Only 16.8% in the coinfected group had serum triglyceride levels below the reference (160 mg/dL). All patients in the HIV-HBV coinfected group had antiretroviral therapeutic agents with dual action anti-HIV and anti-HBV, 87.3% receiving lamivudine alone or in coformulation; in a small number of cases (3.6%) we opted for tenofovir.

## Conclusion

The liver injury in the context of coinfection with HBV remains a key issue in the management of patients with HIV infection and is a major cause of mortality and morbidity.

